# Altered Mitochondrial Protein Homeostasis and Proteinopathies

**DOI:** 10.3389/fnmol.2022.867935

**Published:** 2022-04-27

**Authors:** Aya Jishi, Xin Qi

**Affiliations:** Department of Physiology and Biophysics, Case Western Reserve University School of Medicine, Cleveland, OH, United States

**Keywords:** proteinopathies, protein homeostasis, mitochondrial dysfunction, neurodegeneration, mitochondrial quality control

## Abstract

Increasing evidence implicates mitochondrial dysfunction as key in the development and progression of various forms of neurodegeneration. The multitude of functions carried out by mitochondria necessitates a tight regulation of protein import, dynamics, and turnover; this regulation is achieved *via* several, often overlapping pathways that function at different levels. The development of several major neurodegenerative diseases is associated with dysregulation of these pathways, and growing evidence suggests direct interactions between some pathogenic proteins and mitochondria. When these pathways are compromised, so is mitochondrial function, and the resulting deficits in bioenergetics, trafficking, and mitophagy can exacerbate pathogenic processes. In this review, we provide an overview of the regulatory mechanisms employed by mitochondria to maintain protein homeostasis and discuss the failure of these mechanisms in the context of several major proteinopathies.

## Introduction

The incidence of various neurodegenerative diseases is rapidly growing, and the mechanisms by which many of these diseases develop are not yet completely understood. Though these diseases present differently, there tend to be some commonalities between them. For instance, several exhibit pathological misfolding of proteins that results in their oligomerization and eventual aggregation within or around neurons; these oligomers and aggregates exhibit toxic functions, interfering with various modes of cell function ranging from genomic stability to mitochondrial function (Gan et al., 2018; Ciccocioppo et al., 2020). The clinical features of neurodegenerative diseases can be linked to the neurons primarily affected by these protein aggregates, and the spread of pathological proteins to different brain regions is accompanied by the progression of disease symptoms (Niewold et al., 1987; Drui et al., 2014; Grubman et al., 2019; Masrori and Van Damme, 2020; Wang and Li, 2021). The pathological changes in these proteins can result from increased synthesis, reduced clearance, or post-translational changes; once the initial aggregates are formed, they often recruit and alter proteins, furthering the damaging effects of these structures (Bayer, 2015).

Mitochondria are emerging both as important targets for protein aggregates and as driving forces behind the progression of proteinopathies (Reddy, 2009). Mitochondria can be compartmentalized into two subspaces, the intermembrane space (the region between the outer and inner membranes) and the matrix (the region within the inner membrane). These regions are separated by the inner mitochondrial membrane. Oxidative phosphorylation machinery resides on the inner mitochondrial membrane and is responsible for much of the production of ATP, and a variety of enzymes and proteins reside on the outer mitochondrial membrane, participating in various cellular functions. The role of mitochondria in bioenergetics, biosynthesis, calcium signaling, and oxidative stress regulation, among other pathways, means that accumulating defects in mitochondria contribute to aging and neuronal deterioration (Reddy, 2009; Chistiakov et al., 2014). In proteinopathies, the interactions of key dysfunctional proteins with mitochondria exacerbate these effects, often promoting further oxidative stress, dysregulating respiration machinery, and disrupting energy production (Johri and Beal, 2012). It is becoming apparent that this mitochondrial dysfunction is a key process in disease pathology.

Mitochondria have several, often overlapping pathways that regulate their quality; the buildup of misfolded proteins is normally prevented *via* mitochondrial clearance pathways, or in more extreme situations, dysfunctional mitochondria are degraded in an effort to maintain organelle quality. However, in neurodegeneration, we regularly observe deficits in these quality control mechanisms; often, pathologically misfolded proteins interfere at multiple levels of mitochondrial regulation (Jadiya and Tomar, 2020). Here, we provide a brief overview of these modes of mitochondrial regulation and discuss their dysfunction in the context of several major neurodegenerative diseases.

## Mitochondrial Quality Control Mechanisms

### Protein Import and Folding

The limited scope of mitochondrial DNA necessitates that the majority of mitochondrial proteins should be imported into the organelle. This, coupled with the dense packing of proteins within mitochondria, requires the presence of fine-tuned mechanisms of protein import, folding, and sorting. Mitochondrial protein import is generally post-translational; precursor proteins (still in an unfolded state) containing specific import sequences are formed within the cytosol and then targeted to and transported across the membrane (Dudek et al., 2013). Most commonly, an N-terminal presequence consisting of amphipathic, positively charged, α-helical segments is recognized by the outer membrane receptors Tom20 and Tom70, which are subunits of the translocase of the outer membrane (TOM) complex that selectively bind to different subsets of mitochondrial precursor proteins (Vögtle et al., 2009). Tom20 tends to bind the hydrophobic faces of proteins’ amphipathic α-helical conformations, while Tom70/Tom71 preferentially bind hydrophobic internal targeting sequences (Abe et al., 2000; Saitoh et al., 2007; Dudek et al., 2013). These serve as the first quality control checkpoints of this process; only precursor proteins with the appropriate signaling sequences are allowed through the TOM complex. The main component of the TOM complex is Tom40, which forms a beta-barrel structure embedded in the mitochondrial outer membrane; the other subunits of the complex, including Tom20, Tom70/Tom71, Tom22, Tom5, Tom6, and Tom7, play supportive or regulatory roles (Dudek et al., 2013; Araiso et al., 2019). After recognition, precursor proteins bind to the cytosolic domains of Tom22 and Tom5 and move through the polar pore of the Tom40 barrel, after which they interact with Tom40, Tom7, and Tom22 (Komiya et al., 1998; Rapaport et al., 1998; Dudek et al., 2013).

The insertion of β-barrel proteins into the outer mitochondrial membrane requires additional machinery. These β-barrel proteins contain β-hairpin elements that, when recognized by the TOM complex, allow for their insertion into the outer mitochondrial membrane with the help of the sorting and assembly machinery (SAM) and surrounding chaperones (Höhr et al., 2018). The import of some intermembrane space (IMS) proteins also requires additional machinery (Edwards et al., 2021). Some IMS-directed proteins contain bipartite sequences composed of targeting sequences followed by stop-transfer sequences (Gasser et al., 1982; Burri et al., 2005). These direct proteins toward import, which is then halted at the inner mitochondrial membrane. Sequences are cleaved, releasing mature proteins into the intermembrane space. Other IMS-directed proteins contain characteristic cysteine-rich regions which, after preprotein translocation through the TOM complex, are recognized by the mitochondrial IMS import and assembly (MIA) system (Banci et al., 2009). These regions bind to Mia40, which mediates the formation of disulfide bonds within these peptides, stabilizing them and keeping them from exiting the IMS (Chacinska et al., 2004; Mesecke et al., 2005).

Transport of proteins into the mitochondrial matrix and inner membrane occurs in close cooperation with import through the TOM complex. This transport is mediated by the translocase of the inner membrane 22 (TIM22) and translocase of the inner membrane 23 (TIM23) complexes, which each consist of several subunits and form channels within the inner mitochondrial membrane. TIM22 and TIM23 differ in their protein specificity; TIM23 interacts with positive targeting signals that direct preproteins to the matrix, while TIM22 directs preproteins without classical targeting signals to the inner mitochondrial membrane (Bauer et al., 2000; Jensen and Dunn, 2002). The TIM22 complex contains a channel formed by Tim22 subunits that interact with various other subunits, such as Tim54 and Tim18, which stabilize the complex and mediate protein insertion (Koehler et al., 2000; Kovermann et al., 2002; Peixoto et al., 2007). A group of small proteins in the intermembrane space – including Tim8, Tim9, and Tim10 – help shuttle proteins that have translocated across the TOM complex to TIM22 (Vasiljev et al., 2004; Milenkovic et al., 2007). It is unclear how exactly these subunits operate, but it is suggested that they aid in maintaining secondary structure of precursor proteins and thereby preventing their retrograde movement through the TOM complex. Unlike TIM23, TIM22 responds to internal targeting signals, not N-terminal targeting sequences (Sirrenberg et al., 1998). Though the mechanism of peptide insertion into the inner mitochondrial membrane is still unknown, evidence indicates a dependence of this process on the membrane potential, which is an important feature in mitochondrial function (Peixoto et al., 2007).

The core of the TIM23 complex is composed of two subunits, Tim17 and Tim23, that span the inner membrane and create a channel through which preproteins can move (Zhou et al., 2021). Three subunits of the complex – Tim21, Tim50, and Tim23 – transiently bind to the TOM complex and allow for the immediate translocation of relevant precursor proteins across the inner mitochondrial membrane (Bauer et al., 2000; Tamura et al., 2009). The electric potential of this inner membrane is critical for activation of Tim23. This potential induces an electrophoretic effect that acts on the mostly positively charged presequences of preproteins; this drives their import into the matrix (Voos and Röttgers, 2002; Chacinska et al., 2009; Schmidt et al., 2010; Dudek et al., 2013). Interestingly, mtHsp70, a mitochondrial chaperone protein that can interact with the subunit Tim44, is also essential for inner membrane translocation; this chaperone interacts with precursors as they are translocated across the inner mitochondrial membrane and assists in their folding (Voos and Röttgers, 2002).

Mitochondrial chaperones, often referred to as heat shock proteins, play a range of essential roles in mitochondrial maintenance and protein folding. The size of the translocation pore necessitates that protein precursors be imported in an unfolded or extended state, after which they must be folded into their proper conformations. In addition to their roles in import, heat shock proteins identify unfolded peptides and are the primary facilitators in their folding. Additionally, mitochondrial chaperones promote folding of the mainly hydrophobic oxidative phosphorylation machinery produced by mitochondrial DNA and degrade misfolded proteins, and are therefore crucial in maintaining mitochondrial function and preventing the formation of potentially toxic aggregates (Ryan et al., 1997; Ogilvie et al., 2005). Mitochondrial heat shock protein 70 (mtHsp70) binds to TIM23 and interacts with peptides as they travel through the complex, stabilizing them and increasing folding efficiency (Moro et al., 2002). Another chaperone, Hsp60, forms an oligomeric complex within the mitochondrial matrix that provides a sheltered environment for peptides to fold (Cheng et al., 1989). Similarly, Clp proteins also form oligomeric complexes that resolubilize aggregated proteins (Yu and Houry, 2007).

#### Mitochondrial Ribosomal Quality Control

Synthesis of mitochondrial proteins often occurs near the TOM complex, resulting in a coupling of protein translation and mitochondrial import. At times, translation is stalled, potentially due to mutations or damage in mRNA or defects in ribosomes; when translation is stalled or halted prematurely, the protein fragments generated can contribute to cellular stress, and newly generated peptides are generally targeted for degradation while still associated with the ribosome (Brandman et al., 2012). Ribosomal quality control is driven by Listerin, an E3 ubiquitin ligase that polyubiquitinates polypeptides stalled within ribosomes, and its cofactor nuclear export mediator factor (NEMF) (Martin et al., 2020). Stalled ribosomes are sensed by a few different proteins (HBS1L, GTPBP2, and PELO) and are then split into their large (60S) and small (40S) subunits; the small subunit is recycled and the faulty mRNA is degraded by exoribonucleases in a process known as mitochondrial ribosomal quality control (mitoRQC). The large subunit is recognized by NEMF, a subunit of the ribosomal quality control (RQC) complex, which recruits Listerin, which then induces ubiquitin addition to the translated polypeptide (Bengtson and Joazeiro, 2010). Ubiquitination recruits valosin-containing protein (VCP/p97), which, with the aid of Vms1, extracts nascent chains from stalled ribosomes and delivers them to the proteasome for degradation (Joazeiro, 2019). In certain cases, the close proximity of translating ribosomes to mitochondria, such as in cotranslational import, means these polypeptide chains are not accessible for ubiquitination by VCP/p97. In these cases, Vms1 facilitates mitochondrial quality control by preventing the addition of a C-terminal tail to nascent proteins. Once imported, peptides are degraded by mitochondrial proteases (Izawa et al., 2017). In mammals, ankyrin repeat and zinc finger peptidyl-tRNA hydrolase 1 (ANKZF1), the analog to Vms1, appears to play similar roles in releasing peptides from stalled ribosomes (Verma et al., 2018).

#### Regulation of Protein Import

Defective mitochondrial import allows for the accumulation of precursor proteins on the outer mitochondrial membrane. Unfolded proteins undergo ubiquitination, extraction, and subsequent degradation in a process known as mitochondria-associated degradation (MAD) (Neutzner et al., 2008; Liao et al., 2020). Some studies in yeast suggest that Vms1 recruits Cdc48 to mitochondria in instances of stress, though this is still debated (Heo et al., 2010). Other studies implicate the recruitment of Cdc48 in a related pathway that clears stalled proteins. In yeast, evidence suggests that the membrane protein Ubx2 interacts with the TOM complex and monitors the accumulation of arrested precursor proteins, after which it recruits Cdc48 (VCP/p97 in mammals) to induce proteasomal degradation of these stalled proteins and therefore “unclog” the TOM pore; this is referred to as mitochondrial protein translocation-associated degradation (mitoTAD) (Buchan et al., 2013; Mårtensson et al., 2019).

In a pathway that mirrors both mitoTAD and ribosomal quality control, Vms1 translocates to the mitochondria in instances of cellular stress (Nielson et al., 2017). This protein contains a VCP interaction motif by which it recruits Cdc48 to target proteins on the outer membrane (Hänzelmann and Schindelin, 2011). These proteins are identified *via* ubiquitination by Mdm30. Cdc48 in turn recruits proteasome machinery to initiate degradation (Heo et al., 2010). Recent findings indicate a role for this Cdc48 degradation pathway in regulation of proteins within the mitochondria as well (Liao et al., 2020).

Inhibition of mitochondrial protein import activates a surveillance mechanism that induces a PDR3-mediated transcriptional response. The mitochondrial compromised protein import response (mitoCPR) in yeast is driven by activation of the protein Cis1, which recruits the ATPase Msp1 to Tom70, a subunit of the TOM complex. This initiates the removal and degradation of precursor proteins (Weidberg and Amon, 2018). It is unclear whether this mitoCPR pathway is activated by stalled proteins at the mitochondrial outer membrane or by already translated peptides. The transcriptional changes induced by this response also suggest potential involvement of mitoCPR in other processes, such as restoring redox potential and lipid biosynthesis (Weidberg and Amon, 2018). It is yet to be determined whether this mitoCPR pathway operates in mammalian systems.

When the balance between mitochondrial import and cytosolic capacity is lost, the resulting cytosolic aggregates are disposed of by the mitochondrial precursor overaccumulation stress (mPOS) pathway. Several defects can lead to the toxicity that activates the mPOS pathway, such as dysfunctional protein import machinery, IMM protein misfolding, and a loss of mitochondrial membrane potential (Wang et al., 2008b; Wang and Chen, 2015). The increase in cytosolic stress induces several cellular effects, including altering protein synthesis, inducing degradation, and upregulating translation of stress-resistant proteins (Wang et al., 2008b; Wang and Chen, 2015; Coyne and Chen, 2018). It is important to note that these pathways do not act in isolation. We tend to see interactions between these various forms of mitochondrial protein regulation; several of these pathways involve similar triggers. For example, activation of UPR^mt^ occurs *via* a cytosolic accumulation of protein, which is the inciting factor of mPOS activation. It is possible that these pathways intersect, though that is yet to be proven experimentally.

### UPR^mt^

Accumulation of misfolded or non-functional proteins within mitochondria, *via* dysfunction in mitochondrial translation, for instance, triggers further protein aggregation and proteotoxic stress. The mitochondrial unfolded protein response pathway (UPR^mt^) communicates to the nucleus a need for increased levels of mitochondrial chaperones and proteases, critical factors in proper protein folding and degradation. Gene transcriptional factors that promote synthesis of these chaperones and proteases are activated under stressed conditions in an effort to reestablish proper protein homeostasis (Münch, 2018). Several events can trigger UPR^mt^, including electron transport chain (ETC) impairment, deletion of mitochondrial DNA, and accumulation of reactive oxygen species (ROS) (Muñoz-Carvajal and Sanhueza, 2020).

A key factor in the regulation of UPR^mt^ is activating transcription factor associated with stress 1 (ATFS-1). ATFS-1 contains both a mitochondrial targeting sequence and a nuclear localization signal. Under normal conditions, ATFS-1 is imported into the mitochondria and rapidly degraded by the Lon peptidase LONP1; however, the accumulation of misfolded proteins within mitochondria impairs proper import. This allows for a buildup of ATFS-1 in the cytosol, which then allows for nuclear trafficking. Compromised ATFS-1 import into the mitochondria is sufficient for UPR^mt^ activation and upregulation of protective genes (Nargund et al., 2012). Interestingly, ATFS-1 also appears to promote accumulation of deleterious mtDNA in *Caenorhabditis elegans*, which is associated with aging and various neurodegenerative diseases. ATFS-1 accumulates in dysfunctional mitochondria since its degradation is impaired, where it can associate with deleterious mitochondrial DNA and promote the binding of mtDNA replicative polymerase (POLG) (Yang et al., 2022).

Accumulation of misfolded protein in the mitochondrial matrix induces the transcription of chaperonins *via* activation of the promoter c-Jun N-terminal kinase 2 (JNK2). This induction is mediated in part by the transcription factor C/EBP homologous protein (CHOP), a component of the integrated stress response (ISR) pathway, though activation likely occurs through a separate pathway than in the integrated stress response (Horibe and Hoogenraad, 2007). CHOP alone is not sufficient for chaperonin induction; several other factors, including the two promoter elements mitochondrial unfolded protein response elements 1 and 2 (MURE1 and MURE2), allow for adequate and specific signaling (Aldridge et al., 2007; Münch, 2018). ATF5, another CHOP target, carries both a nuclear targeting sequence and a mitochondrial targeting sequence; mitochondrial stress drives increased nuclear localization and consequently upregulation of UPR^mt^ genes (Fiorese et al., 2016). Heat shock protein and mitochondrial protease induction can be accompanied by a decrease in mitochondrial protein import and translation, and hence a reduced folding load, as well as an increase in antioxidant activity *via* sirtuins, lysine deacetylases and ADP-ribosyltransferases that drive expression of FOXO3A, a transcription factor coding for antioxidant enzymes (Mouchiroud et al., 2013; Münch, 2018).

Accumulation of proteins in the intermembrane space can initiate an alternate UPR^mt^ pathway by causing phosphorylation of the protein kinase AKT, which then causes estrogen receptor α (ERα activation). The transcription factor NFR1 is upregulated, which in turn upregulates expression of nuclear and mitochondrial genes contributing to mitochondrial respiration. Additionally, the IMS protease HtrA Serine Peptidase 2 (HTRA2) is upregulated, leading to increased proteasomal activity. In another CHOP-independent pathway, mitochondrial stress results in the activation of sirtuin 3, which modulates the expression and activity of the superoxide dismutases (SODs) and regulates antioxidant mechanisms and mitophagy (Papa and Germain, 2014).

### Mitochondrial Proteases

Protein import into the mitochondria also undergoes mitoprotease regulation; mitochondrial processing peptidases, for instance, cleave targeting and sorting sequences from newly imported proteins, thereby allowing them to mature functionally. Additional processing is necessary for stabilizing certain proteins, such as MRPL12 (Nouws et al., 2016). This maturation is primarily mediated by the mitochondrial processing peptide (MPP), which cleaves mitochondrial targeting signals from peptides and allows them to mature. Cleaved mitochondrial targeting sequences are then fragmented by the metalloprotease PrEP (Kücükköse et al., 2021). The cleaved signaling peptides are further degraded into amino acids by oligopeptidases in the matrix and intermembrane space. These processing mechanisms appear to play additional regulatory roles in localization and activity of mitochondrial proteins, and defects in these cleavage pathways are associated with the formation of aggregates of unprocessed proteins (Zurita Rendón and Shoubridge, 2018; Deshwal et al., 2020).

Mitoproteases further modulate mitochondrial import *via* altering the functionality of import machinery. The *i*-AAA protease YME1-like 1 (YME1L) degrades a subunit of the TIM23 complex, TIMM17A, impeding mitochondrial import and reducing mitochondrial load (Rainbolt et al., 2013). Certain proteases, such as Lon peptidase 1 (LONP1) and ClpXP in the mitochondrial matrix, hydrolyze ATP to unfold and translocate proteins into their proteolytic chamber for degradation (Lee et al., 2021). ClpP additionally exerts chaperone-like activity, while some AAA proteases interact with membrane scaffolds to define localized subregions of proteolytic activity, contributing to mitochondrial compartmentalization (Deshwal et al., 2020).

The *m*-AAA protease is key in regulating and degrading misassembled and damaged proteins on the inner mitochondrial membrane. The AAA proteases are defined by an AAA sequence, an ATPase associated with various cellular activities, and a proteolytic domain facing the mitochondrial matrix (as opposed to *i*-AAA proteases, which contain a proteolytic domain exposed to the intermembrane space). These AAA proteases extract transmembrane segments embedded in membranes and proteolytically degrade them into smaller peptides, which can then be further broken down into amino acids or exported from the organelle (Martinelli and Rugarli, 2010). Additionally, these *m*-AAA proteases, *via* proteolytic degradation, are responsible for maturing certain proteins, including the mitochondrial ribosomal component MRPL32 (Bonn et al., 2011). As such, reduced *m*-AAA levels or defects in activity can cause reduced protein synthesis and eventually defective respiration.

### Mitochondrial Dynamics and Mitophagy

Fission is mediated by cytoplasmic Drp1 and the outer mitochondrial membrane protein Fis1, while fusion of the outer mitochondrial membranes (OMM) is mediated by mitofusins 1 and 2 (Mfn1 and Mfn2), and fusion of the inner mitochondrial membranes (IMM) is mediated by optic atrophy protein 1 (OPA1) (Zuo et al., 2020). Early in the process of fission, the mitochondrial membrane comes into contact with the ER, creating structures called mitochondria associated membranes (MAMs), and various proteins are recruited to the junctions. Fission begins with the recruitment of Drp1 from the cytosol; phosphorylation of Drp1 causes its oligomerization, and the recruited proteins essentially form ring-like structures around mitochondria that constrict to separate the membranes (Youle and Van Der Bliek, 2012; Yang et al., 2021). Evidence suggests that nucleoid separation between fragments occurs *via* a yet poorly understood mechanism mediated by mitochondrial contact site and cristae organizing system (MICOS) and Miro1 (Yang et al., 2021). Fusion requires that two membranes be brought close together, achieved using attachment protein receptors with coiled-coil domains that interact with one another, and deformation of the lipid membranes, achieved using hydrophobic domains inserted into the membranes (Westermann, 2010). Fusion of the outer OMM occurs first and is mediated by Mfn1 and Mfn2, which are GTPases, followed by fusion of the IMM, which is mediated by OPA1 (Yang et al., 2021).

Recently, mitochondrial proteases have emerged as key players in mitochondrial lipid metabolism and fission/fusion dynamics. Proper lipid synthesis and distribution relies on trafficking of lipids, both from the endoplasmic reticulum to the mitochondria and between mitochondrial membranes. Certain lipid transfer proteins in the intermembrane space, specifically those of the PRELID/Ups family, are continuously degraded by the *i*-AAA protease YME1L (Potting et al., 2010; Miliara et al., 2015). These proteins interact with TP53 regulated inhibitor of apoptosis 1 (TRIAP1), and shuttle phosphatidic acid or phosphatidylserine across the IMS (Miliara et al., 2015). The metabolism of these proteins therefore impacts transport rates of these lipids, and therefore the rates of synthesis of phosphatidylethanolamine and cardiolipin. Similarly, degradation of fusion-competent L-OPA1 regulates mitochondrial fragmentation and consequently mitophagy (Deshwal et al., 2020).

Mitophagy refers to the autophagic process of degrading damaged or dysfunctional mitochondria; this process is a key mechanism in maintaining overall mitochondrial quality by clearing out unhealthy organelles. Various stimuli can trigger mitophagy through two main pathways: PINK1/Parkin-mediated mitophagy and receptor-mediated mitophagy. Under normal conditions, PINK1 is continuously transported into mitochondria in a mitochondrial membrane potential dependent manner, where its transmembrane domain and mitochondrial targeting sequence are cleaved, and the protein is degraded (Nguyen et al., 2016). However, in cases of mitochondrial damage, PINK1 import is disrupted as a result of either decreased membrane potential or accumulation of misfolded proteins in the mitochondrial matrix. As such, PINK1 dimers begin to form at the TOM complex and accumulate on the outer mitochondrial membrane. PINK1 dimers undergo autophosphorylation and phosphorylate ubiquitin, which recruits and activates Parkin, and E3 ligase. Parkin polyubiquitinates the mitochondria, tagging them for autophagic degradation (Martinez-Vicente, 2017; Zuo et al., 2020).

Mitophagy can also be induced *via* activation of certain mitophagy receptors, such as FUN14 domain containing 1 (FUNDC1) on the outer mitochondrial membrane and prohibitin-2 (PHB2) on the inner mitochondrial membrane (Wei et al., 2017). Various stimuli activate these receptors, initiating pathways that eventually lead to binding of the affected mitochondria to LC3 and degradation. For example, under normal physiological conditions, FUNDC1 is phosphorylated and hence suppressed; however, in hypoxic conditions, FUNDC1 is dephosphorylated, disrupting its association with OPA1 and preventing fusion (Liu et al., 2012; Chen et al., 2016). Additionally, it recruits Drp1 to mitochondria-ER contact sites, inducing fission, and binds to LC3, initiating mitophagy (Wu et al., 2016). PHB2, which is located on the inner mitochondrial membrane, responds to stresses such as membrane depolarization by stabilizing PINK1 on the OMM and eventually leading to proteasome-dependent outer mitochondrial membrane rupture (Yan et al., 2020). The externalized PHB2 also interacts with LC3 directly to trigger organelle degradation (Xiao et al., 2018).

The mechanisms regulating mitochondrial dynamics, namely fission and fusion, overlap strongly with the mechanisms of mitophagy. In addition to its role in mitochondrial fusion, Mfn2 contributes to the attachment of mitochondria to the ER and hence the creation of MAMs. In stressed cellular conditions, Mfn2 is ubiquitinated and Drp1 is recruited to these MAMs, dissolving them and causing mitochondrial fission (de Brito and Scorrano, 2009; Basso et al., 2018). Though it is not sufficient to induce mitophagy, fragmentation is an important early event in the process, as fragmented mitochondria can be classified as either polarized or unpolarized. Unpolarized mitochondria are targeted for mitophagy (Xiong et al., 2019). Drp1 deficient mouse models exhibit enlarged mitochondria and increased MPTP-associated mitophagy, while Mfn2 overexpression promotes mitophagy *via* Parkin translocation and phosphorylation (Xiong et al., 2019; Zuo et al., 2020).

## Mitochondrial Quality Control Mechanisms in Proteinopathies

**FIGURE 1:**
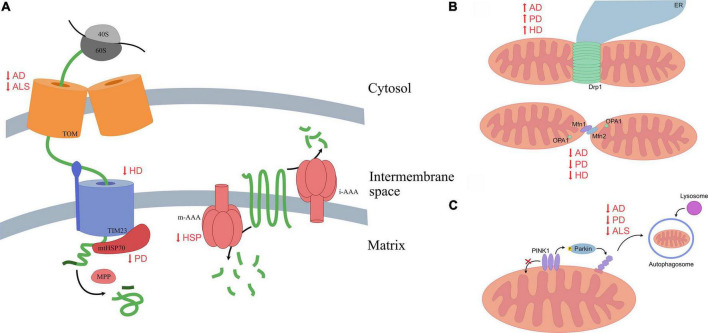
A summary of altered mitochondrial mechanisms in multiple neurodegenerative diseases. **(A)** Normally, peptides targeted for the mitochondrial matrix move through the TOM complex on the outer mitochondrial membrane, then through the TIM23 complex, where mitochondrial chaperones assist in proper folding and processing enzymes cleave peptides, allowing them to mature. Various proteinopathies alter efficiency of mitochondrial protein import at either the outer or inner membrane, while others are characterized by reduced protease activity. Impaired import results in protein aggregation within mitochondria or the surrounding cytosol, chronically activating mitochondrial quality control mechanisms. **(B)** Mitochondrial fission is mediated by oligomers of Drp1 that constrict the organelle; fusion of the outer mitochondrial membranes is mediated primarily by Mfn1 and Mfn2, while fusion of the inner mitochondrial membrane is mediated by OPA1. A common characteristic of these neurodegenerative diseases is an increase in fission and a decrease in fusion. This leads to fragmentation of mitochondria, reducing their efficiency. **(C)** Mitophagy is commonly inhibited in proteinopathies; this leads to accumulation of damaged mitochondria.

Mitochondrial dysfunction is emerging as a common theme in various proteinopathies, especially proteinopathies affecting neuronal function. Cellular stress, energy production deficits, and impaired mitochondrial trafficking result in increased neuronal vulnerability and contribute to the processes that bring about degeneration. Normally, the various modes of mitochondrial quality control allow for the maintenance of mitochondrial health, or in cases where function cannot be rescued, the clearance of damaged mitochondria. However, in various proteinopathies, pathogenic mutations in key proteins result in aggregation of misfolded proteins, which can cause cellular damage through interference with multiple pathways. Accumulating evidence implies both direct and indirect associations of these pathogenic proteins with mitochondria, and in several cases, they directly inhibit the ability of quality control mechanisms to maintain mitochondrial health. Mitochondrial damage then exacerbates the effects of these misfolded proteins, resulting in an increasingly toxic feed-forward loop that eventually results in neurodegeneration (Briston and Hicks, 2018). Here, we summarize (see Figure 1 and Table 1) and discuss the impact of altered mitochondrial quality control on several neurodegenerative diseases, focusing on the interplay between these mechanisms and the characteristic proteins of these diseases. We find that in many cases, the issue of causality in these relationships is unclear; often, we find that the accumulation of pathological proteins and mitochondrial dysfunction form a cycle of increasing severity. Identifying the initial mode of dysregulation is difficult, but as the diseases progress, protein accumulation induces progressive mitochondrial damage, which in turn allows for greater protein aggregation.

**TABLE 1:** A summary of mitochondrial protein homeostasis dysregulation in neurodegeneration.

Proteinopathy	Key proteins	Protein functions	Effects on mitochondrial quality control
Alzheimer’s disease	Amyloid β	Several potential roles, including kinase activation and protection against oxidative stress	Oligomerization of Aβ affects mitochondrial import machinery, causing preprotein accumulation and UPR^mt^ activation. Mitochondrial dynamics and mitophagy are also impaired, and microglia are activated, causing increased release of inflammatory cytokines (Wang et al., 2008a; Heneka et al., 2015; Beck et al., 2016)
	Tau	Microtubule stabilization and regulating axonal transport	Hyperphosphorylated tau causes microtubule instability that affects cellular transport. It also interacts with Drp1, and along with decreased Mfn1/2 levels, causes mitochondrial fragmentation (Gong and Iqbal, 2008; Kandimalla et al., 2016)
Parkinson’s disease	α-synuclein	Synaptic vesicle processing and neurotransmitter release	Mutant α-synuclein interacts with the TOM complex and disrupts import. It also recruits Drp1 to mitochondria, increasing fission, and suppresses mitochondrial protease activity (Franco-Iborra et al., 2018; Vicario et al., 2018)
	LRRK2	Phosphorylation of various substrates and GTP-GDP hydrolysis	Increased phosphorylation of Drp1 and interference with the Parkin-Drp1 interaction promotes increased mitochondrial fragmentation (Su and Qi, 2013; Bonello et al., 2019)
	PINK1/Parkin	Regulation of mitophagy	Reduced levels of these proteins impair mitophagy and inhibits UPR^mt^ initiation (Franco-Iborra et al., 2018)
	mtHsp70	Mitochondrial precursor import and folding	Reduced levels in PD impair proper protein folding, induce oxidative stress, and may affect mitophagy (Burbulla et al., 2010; Klein et al., 2011)
Huntington’s disease	Huntingtin	Axonal transport and regulation of autophagy	Gain-of-function mutations affect mitochondrial import, enhance Drp1 activity *via* S-nitrosylation, and affect mitophagy (Haun et al., 2013; Guo et al., 2016; Franco-Iborra et al., 2021)
	ATAD3A	Regulation of mitochondrial dynamics and stabilization of nucleoids	Increased dimerization of ATAD3A in HD results in greater Drp1-mediated mitochondrial fission (Guo et al., 2013; Zhao et al., 2019)
Amyotrophic Lateral Sclerosis	TDP-43	Transcriptional repression and translational regulation	Accumulation within mitochondria causes prolonged UPR^mt^ activation (Wang et al., 2019)
	SOD1	Elimination of superoxide radicals	Accumulation within mitochondria causes prolonged UPR^mt^ activation and oxidative damage (Gomez and Germain, 2019)
	TBK1/Optineurin	Regulation of mitophagy	Mutations in these proteins disrupt mitophagy and cause accumulation of dysfunctional mitochondria (Granatiero and Manfredi, 2019)
Hereditary spastic paraplegia	Paraplegin	Component of m-AAA protease	Reduced paraplegin levels affect ribosomal processing and degradation of membrane proteins (Ferreirinha et al., 2004; Maltecca et al., 2008; Martinelli and Rugarli, 2010)
Spinocerebellar ataxia	AFG3L2	Component of m-AAA protease	Reduced levels affect m-AAA enzyme activity, causing enlarged mitochondria, disrupted cristae, and impaired axonal trafficking (Martinelli and Rugarli, 2010; Smets et al., 2014)

### Alzheimer’s Disease

Alzheimer’s disease (AD) is the most common neurodegenerative disease and is characterized by progressive dementia and loss of cognitive function due to neurodegeneration, primarily observed in the cortex and hippocampus. Commonly, β-amyloid (Aβ) protein, which is derived from cleavage of amyloid precursor protein (APP), forms deposits extracellularly. Deposits of tau protein, known as neurofibrillary tangles, are also observed within cells. Mutations in APP, specifically mutations resulting in increased Aβ levels, are common in inherited forms of AD, and one of the resulting isoforms, Aβ42, is more prone to formation of these pathogenic plaques. Additionally, AD patients frequently exhibit decreased rates of Aβ clearance (Breijyeh and Karaman, 2020; Knopman et al., 2021). Here, we discuss the changes in UPR^mt^, mitochondrial clearance and dynamics, and neuroinflammation observed in AD.

Aggregation of amyloid beta peptides in AD is often observed within mitochondria. Translationally arrested, non-glycosylated APP tends to accumulate within the protein import machinery of mitochondria in AD patients, physically inhibiting the passage of peptides into the organelle. Additionally, APP associates and forms complexes with the TOM and TIM complexes, exacerbating the negative effects that on mitochondrial function (Devi et al., 2006). These include reducing mitochondrial membrane potential and therefore energy production ability, increasing hydrogen peroxide accumulation, and allowing for the accumulation of preproteins as a result of impaired protein import (Palmer et al., 2021). This buildup of protein can then activate UPR^mt^, which may play a protective role with regards to APP-mediated toxicity (Shen et al., 2020). Analysis of postmortem samples of frontal cortices of AD patients found notable upregulation of UPR^mt^ genes, though it is unclear if this alteration to UPR^mt^ function occurs early in AD pathogenesis or as a late stage response to cellular degeneration (Beck et al., 2016).

Overexpression of APP causes an increase in fission, leading to the accumulation of fragmented mitochondria (Wang et al., 2008a). Additionally, hyperphosphorylation of tau, one of the characteristic proteins of AD, causes microtubule instability (Michaelis et al., 2002). In *C. elegans* models, tau-induced changes in mitochondrial function are observed early in the course of tauopathy; evidence suggests a relationship between mitochondrial dysfunction and calcium imbalance in these systems (Palikaras et al., 2021). Moreover, oligomeric forms of tau have been found in the hippocampus early in the course of disease development in mouse models of tauopathy (Zheng et al., 2020). This change is accompanied by the presence of elongated mitochondria (indicative of altered mitochondrial dynamics) and altered levels of key mitochondrial proteins, including PINK1 and Drp1. These changes, along with a decrease in ATP production rates, impair proper mitochondrial transport and distribution, and cause synaptic damage, reduced neuronal connectivity, and eventually cell death (Gong and Iqbal, 2008; Alonso et al., 2018). Phosphorylated tau also appears to interact with Drp1, increasing mitochondrial fragmentation; a potential interaction with amyloid beta exists as well, though the pathways this interaction is involved in, as well as interactions between tau, amyloid beta, and Drp1, are not well understood (Kandimalla et al., 2016). As AD progresses, levels of Mfn1 and Mfn2 decrease in neurons, also suggesting a decrease in fusion and an overall breakdown of normal mitochondrial dynamics (Manczak et al., 2016; Yang et al., 2021).

A key mitophagy pathway, the PINK1/Parkin pathway, is also affected in AD patients, with PINK1 levels being lower in AD patients than in unaffected patients (Du et al., 2017). Additionally, accumulating amyloid beta further impairs mitochondrial clearance. As mitophagy cannot occur normally, dysfunctional mitochondria begin to accumulate within cells (Vaillant-Beuchot et al., 2021). However, it is still unclear whether these pathways are early drivers of disease or whether mitochondrial and autophagic dysfunction caused by aggregate formation eventually lead to mitophagic disruption. Recovery of mitophagic function *via* NAD^+^ supplementation, PINK1 overexpression, urolithin A, or actinonin recovers some aspects of AD pathology, such as reducing levels of insoluble amyloid beta, reducing levels of hyperphosphorylated tau, and preventing cognitive impairment (Du et al., 2017; Fang et al., 2019). Small molecule mitophagy inducers similarly increase functionality of glutamatergic and cholinergic neurons, and reduce protein aggregation (Xie et al., 2022).

Prolonged neuroinflammation is a common feature of AD as well as several other neurodegenerative diseases. Aβ oligomers and fibrils bind to receptors on microglia and activate them, causing cytokine and chemokine production, which causes inflammation. Normally, microglia engulf and degrade Aβ, but in AD, this degradation is impaired, allowing for Aβ accumulation (Heneka et al., 2015). This, coupled with the release of pro-inflammatory cytokines, which are involved in Aβ aggregation, results in prolonged neuroinflammation (Sastre et al., 2008). One consequence of effective mitochondrial clearance is the reduction of secreted inflammatory cytokines. In other words, mitophagy allows for a reduced inflammatory response, which likely reduces neuronal damage (Fang et al., 2019; Yang et al., 2021). Mice expressing mutant APP are reported to have reduced levels of NAD^+^ and increased levels of inflammation markers. This NAD^+^ depletion activates the cGAS-STING pathway, contributing to neuroinflammation (Hou et al., 2021). In AD patients, increased mitochondrial fragmentation results in release of mitochondria into the extracellular space, inducing a greater immune response, and inhibition of mitochondrial fission reduces expression of neurotoxic proteins (Joshi et al., 2019). When autophagy is compromised, release of mitochondrial DNA causes increased production of certain inflammatory cytokines (Moya and Rivera, 2021). The increase in ROS levels also activates signaling cascades that induce greater cytokine production. These studies suggest that impaired mitochondrial dynamics and inhibited mitophagy in AD contribute to immune activation and its pathogenic consequences in multiple ways.

### Parkinson’s Disease

Parkinson’s disease (PD) is a multifactorial disease characterized by a progressive degeneration of motor ability due to a loss of dopaminergic (DA) neurons in the substantia nigra. Mutations in some key proteins, such as α-synuclein and leucine-rich repeat kinase 2 (LRRK2), are associated with heritable forms of the disease, but incidence of PD is largely sporadic. Pathogenesis is influenced by various factors, including toxin exposure, the gut microbiome, and genetic factors, and is distinguished by the presence of Lewy bodies, which are aggregates composed primarily of α-synuclein (Chen et al., 2019). The function of α-synuclein under normal conditions is not completely understood; it is involved in vesicle dynamics, and some evidence suggests a role for α-synuclein in mitochondrial function, but the exact mechanisms of its action are unclear. Like in AD, mitochondrial protein accumulation alters UPR^mt^, mitophagy, and mitochondrial dynamics, though the specific mechanisms by which this occurs vary.

Changes to mitochondrial import and folding can disrupt the balance of nucleus- to mitochondria-derived proteins within the organelle. The function of chaperone proteins is therefore critical in maintaining proper import and preventing the accumulation of unfolded or misfolded peptides. The accumulation of α-synuclein is a defining characteristic in Parkinson’s disease; in PD patients, increased levels of α-synuclein are observed within mitochondria, though it is still unclear how exactly this protein interacts with existing mitochondrial machinery (Chen et al., 2019). Current evidence suggests that α-synuclein interacts with Tom40, Tom20, and Sam50, components of the mitochondrial protein import machinery (McFarland et al., 2008; Bender et al., 2013; Di Maio et al., 2016). Some studies indicate a disruption of the Tom20-Tom22 interaction due to α-synuclein; other studies show reduced Tom40 levels in postmortem PD patient samples (Bender et al., 2013; Di Maio et al., 2016). These studies suggest that altered mitochondrial import machinery allows for the increased rate of α-synuclein entry and accumulation, though the exact modes through which this occurs are unclear. Interestingly, mutant forms of α-synuclein appear to affect protein import machinery *in vitro* (Devi et al., 2006; Franco-Iborra et al., 2018; Vicario et al., 2018).

Additionally, levels of mtHsp70, and important chaperone involved in mitochondrial import and folding, are decreased in the substantia nigra of PD patients. Mutations in *HSP9*, the gene encoding mtHsp70, were found in a small subset of PD patients, suggesting a potential connection between altered function of chaperones and the onset and progression of PD. These mutants are associated with mitochondrial dysfunction and increased oxidative stress (Burbulla et al., 2010, 2014). The expression of unfolded proteins within mitochondria – as a result of chaperone of protease dysfunction, for instance – causes accumulation of PINK1 on energetically viable mitochondria, inducing mitophagy unnecessarily and reducing availability of functional mitochondria (Klein et al., 2011; Franco-Iborra et al., 2018). Interestingly, PD is associated with impaired mitophagy *via* reduced PINK1/Parkin activity, and it is unclear how this induction of mitophagy *via* PINK1 accumulation fits into the picture (Miller and Muqit, 2019).

Mitophagic induction *via* unfolded protein accumulation suggests some crosstalk between mitophagy and UPR^mt^ (Jin and Youle, 2013; Muñoz-Carvajal and Sanhueza, 2020). Loss of PINK1 and Parkin results in accumulation of damaged mitochondria due to a lack of adequate clearance, eventually resulting in neuronal degeneration. Downregulation of PINK1 and PDR-1 (Parkin ortholog) in *C. elegans* activates UPR^mt^; this activation serves to mitigate the effects of mitochondrial dysfunction and decrease the rate of dopaminergic neuron degeneration (Cooper et al., 2017). Therefore, it stands to reason that preventing the initiation of UPR^mt^, for instance *via* loss of functional ATFS-1, increases mitochondrial sensitivity to stress and decreases overall cellular lifespan.

The accumulation of mutant A53T α-synuclein in the mitochondria is a hallmark of certain familial forms of PD. Within the mitochondria, it interacts with ClpP, a mitochondrial protease and regulator of UPR^mt^, and suppresses its peptidase activity. Overexpression of either wild type or mutant α-synuclein is associated with decreased ClpP levels, and this impairs respiration, inhibits bioenergetics, induces oxidative stress by reducing SOD2 activity, and reduces clearance of unfolded and misfolded proteins (Hu et al., 2019). The resulting accumulation of proteins within the mitochondria activates UPR^mt^, and though short-term activation can prove useful in protein clearance, prolonged activation can have negative effects, allowing for the accumulation of dysfunctional mitochondria.

Protein degradation in the mitochondrial matrix is mediated in part by the protease LONP1; certain PD mouse models exhibit increased LONP1 levels, potentially as a result of an increase in levels of oxidized proteins and ROS. The increase in ROS levels, however, inactivates LONP1 (Bulteau et al., 2017). Another protease found in the mitochondria, HTRA2, has also been implicated in PD. PD-linked mutations in this protease impair its activity, and a potential link with PINK1 pathways may connect this protein to major PD pathogenic mechanisms (Plun-Favreau et al., 2007).

The most common causes of familial PD are mutations in the kinase LRRK2; several of these increase kinase activity, phosphorylating Drp1 and causing mitochondrial fragmentation (Su and Qi, 2013). LRRK2 also interferes with the Parkin-Drp1 interaction, and affects mitochondrial transport and degradation (Bonello et al., 2019). Mitochondrial dynamics are altered in dopaminergic neurons in PD patients; mutant α-synuclein localizes to mitochondria and causes Drp1 recruitment and therefore increased mitochondrial fragmentation, which eventually contributes to dysfunction and reduced energy output. Furthermore, like in AD, the PINK1/Parkin mediated mitophagic pathway is impaired in PD, again leading to an accumulation of dysfunctional mitochondria, limited autophagy, and exacerbated accumulation of proteins in cells that lead to further disruption of key cellular functions (Narendra et al., 2008; Clark et al., 2021). Parkin activation is regulated by the state of ubiquitination, and the interplay of ubiquitinating and deubiquitinating enzymes (DUBs) is key in regulating mitophagy. Some evidence indicates that these DUBs also deubiquitinate α-synuclein, contributing to its aggregation and negative effects (Yang et al., 2021). Additionally, treatment with urolithin A, which promotes mitophagy, recovers mitochondrial function and motor deficits in the 6-OHDA treated PD mouse model. Urolithin A promotes mitochondrial biogenesis *via* the SIRT1-PGC-1α pathway and attenuates the inflammatory response in microglia by inhibiting NLRP3 inflammasome activation (Kujawska et al., 2020; Liu et al., 2022; Qiu et al., 2022). These findings again pointing to the dysregulation of mitochondrial dynamics and clearance as prominent causes of PD pathology.

### Huntington’s Disease

Huntington’s disease (HD) is a neurodegenerative disease where patients exhibit cognitive, motor, and psychiatric changes. The loss of medium spiny neurons causes first a period of hyperkinesia, characterized by frequent involuntary movement, followed by a hypokinetic phase, characterized by bradykinesia and gait disturbance. Neuropsychiatric symptoms include anxiety, obsessive compulsive behavior, psychosis, and apathy (McColgan and Tabrizi, 2018). At the cellular level, HD is caused by an expansion of the CAG repeat in exon 1 of the huntingtin gene, with a greater number of repeats typically correlating with earlier onset of disease symptoms (Tabrizi et al., 2020).

The protein huntingtin is not yet well-understood, but appears to shuttle between the nucleus and cytosol and interact with a large number of proteins, including transcription factors, and changes to these interactions may drive disease pathogenesis. The prevailing theory is mutations in huntingtin convey protein gain of function in HD; this leads to protein aggregation (Bossy-Wetzel et al., 2008; Tabrizi et al., 2020). The localization of mutant huntingtin to mitochondria appears to affect mitochondrial import machinery, specifically the TIM23 complex. Impaired mitochondrial import is found early in development of disease, especially in cells with high energetic demands, such as medium spiny neurons (Quintanilla and Johnson, 2009).

Like other neurodegenerative diseases, HD patients exhibit reduced levels of Mfn1, Mfn2, and OPA1, and increased levels of Drp1. Mutant huntingtin, the driving protein behind HD, appears to enhance the activity of Drp1 localized on mitochondria. Mutant huntingtin causes overproduction of nitric oxide, which interacts with Drp1, leading to its S-nitrosylation and increased fission. Introduction of non-nitrosylatable versions of huntingtin partially rescue the damage done to mitochondria and overall cellular health in HD models (Haun et al., 2013). ATPase family AAA-domain containing protein 3A (ATAD3A) has also been linked to Drp1 hyperactivation; ATAD3A deacetylation causes its dimerization, which is required for Drp1-mediated fission. This dimerization increases in HD, along with increased ATAD3A localization at mitochondrial contact sites. This gain of function in ATAD3A in turn induces mitochondrial fragmentation and impairs mtDNA maintenance, contributing to pathology (Zhao et al., 2019). The inhibition of mitochondrial fragmentation *via* inhibition of Drp1 improves cell viability and rescues some HD phenotypes in animal models (Guo et al., 2013).

Huntingtin participates in the regulation of several intracellular processes, including vesicle transport and autophagy. Huntingtin acts as a scaffolding protein, bringing together the various proteins to create the complexes necessary for mitophagy. Changes to the huntingtin protein in turn change its ability to properly interact with its recruited proteins, destabilizing the resulting complexes. Cells expressing mutant huntingtin exhibit inefficient mitochondrial degradation, in part because the polyglutamine stretches in mutant huntingtin affects the interactions between LC3-II and mitophagy receptors (Franco-Iborra et al., 2021). However, various models have shown an interaction between VCP and mutant huntingtin, with mutant huntingtin recruiting VCP to mitochondria. The accumulation VCP causes increased mitophagy through interactions with LC3, and the excessive degradation of mitochondria may result in a deficit in functional mitochondria (Guo et al., 2016).

### Amyotrophic Lateral Sclerosis

Amyotrophic lateral sclerosis (ALS) presents as progressive muscle weakness and eventually paralysis that comes about as a result of motor neuron degeneration. Patients experience highly variable ages of onset and rates of progression, and disease manifestation is often sporadic, with multiple factors contributing to disease development. Symptoms tend to have a focal point of onset, but spread over time, leading to motor dysfunction, language disruption, and respiratory issues. Like PD, most cases of ALS are sporadic, though about 10% of cases are familial. At the cellular level, ALS is characterized by formation of aggregates in motor neurons, typically composed mainly of TDP-43, that disrupt various aspects of cell function (Hardiman et al., 2017; Masrori and Van Damme, 2020).

The protein CHCHD10 has been linked to ALS, and though its function is not yet completely understood, it appears to localize to the intermembrane space and to associate with the mitochondrial contact site and cristae organizing system (MICOS). In some cases of ALS, mutations in CHCHD10, specifically some mutations in the coiled-coil-helix-coiled-coil-helix domains of the proteins, cause defects in the mitochondrial import of the protein. This results in clustering of protein in the cytoplasm or within the mitochondria (Lehmer et al., 2018; Tazelaar et al., 2018).

Mitochondrial dysfunction, specifically that caused by the dysfunction of SOD1 or TDP-43, is a hallmark of ALS, and mutant forms of these proteins tend to collect in the intermembrane space of the mitochondria, triggering UPR^mt^ *via* two of its distinct mechanisms of activation. SOD1 is responsible for converting superoxide radicals into oxygen and hydrogen peroxide, and oxidative damage resulting from defective SOD1 function likely causes mitochondrial damage that contributes to pathogenesis and disease progression (Gomez and Germain, 2019; Wang et al., 2019). TDP-43 is involved in transcriptional repression and translational regulation. Evidence suggests that the CHOP-dependent and Akt-dependent pathways are activated both before and throughout progression of ALS. Consistent overactivation of these pathways eventually results in pathology and increased cellular stress.

Mild levels of cellular stress cause recruitment of optineurin (OPTN) to damaged mitochondria, initiating OPTN-mediated mitophagy (Weil et al., 2018). The loss of mitochondrial membrane polarization or generation of ROS causes a Parkin-dependent recruitment of OPTN, which binds to ubiquitinated mitochondria *via* a ubiquitin-binding domain. In neurons, this process occurs primarily in the soma. OPTN contains an LC3 interaction region that induces autophagosome formation. Mitochondria are engulfed in mitophagosomes and degraded, though the process of their degradation is rather inefficient due to the slow acidification of these structures (Liu et al., 2018; Evans and Holzbaur, 2020).

Binding of optineurin to these mitochondrial proteins is mediated by Tank-binding kinase (TBK1), which phosphorylates it and therefore encourages mitophagy. Mutations in both optineurin and TBK1 are found in familial forms of ALS; in these cases, mitophagy is disrupted, and dysfunctional mitochondria accumulate within cells (Millecamps et al., 2011). Expression of an ALS-linked OPTN mutation causes a decrease in mitochondrial membrane potential and inhibited mitophagy, which keeps damaged mitochondria within the cellular mitochondrial network, reducing overall function. The resulting increase in damaged mitochondria further exacerbates the issue, as OPTN is unable to adequately sequester them, leading to a cycle of increasing severity of dysfunction (Wong and Holzbaur, 2014). Mutation of VCP, another mitochondrial quality control protein that extracts stalled proteins, is also associated with ALS and is associated with impaired clearance of damaged organelles (Granatiero and Manfredi, 2019).

### Hereditary Spastic Paraplegia and Spinocerebellar Ataxia

Hereditary spastic paraplegia (HSP) is characterized by progressive spasticity and weakness of the lower body that arises as a result of cortical motor neuron axonal degeneration (Martinelli and Rugarli, 2010; Gumeni et al., 2021). HSPs are clinically and genetically diverse, but are classified by degeneration of the long nerve fibers in the corticospinal tracts and posterior columns. Manifestation of symptoms is gradual, beginning with stiffness of the leg or sensory abnormalities and progressing to ataxia and amyotrophy, among other symptoms. Autosomal dominant, autosomal recessive, and X-linked forms of HSP have been identified, resulting in clinical several manifestations of the disease (Salinas et al., 2008).

Though HSP is heterogeneous, loss of function mutations in paraplegin, a subunit of the m-AAA protease, have been found to be associated with certain familial cases and some sporadic cases of HSP (Banfi et al., 1999). This loss of paraplegin implies a dysfunction of the hetero-oligomeric paraplegin-AFG3L2 (AFG3 ATPase family gene 3-like 2) complex, which processes the ribosomal component MrpL32 and degrades non-native membrane proteins. In Spg7^–/–^ knockout mouse models (i.e., mice lacking paraplegin), age brings about abnormal movement of the hindlimbs, scoliosis, and late onset axonal degeneration in motor spinal tracts, phenotypes that resemble HSP (Atorino et al., 2003).

Similarly, spinocerebellar ataxias are a heterogeneous group of autosomal dominantly inherited diseases, often characterized by progressive gait and limb ataxia as well as a loss of balance caused by degeneration of the cerebellum and its connecting neurons. Purkinje fibers, the spinal cord, and basal ganglia are often implicated in spinocerebellar ataxias as well. The clinical heterogeneity of these diseases complicates therapeutic development, and current treatments focus on symptom management (Klockgether et al., 2019).

The genetic locus containing AFG3L2 has been mapped as an important region in both familial and sporadic forms of cerebellar ataxia. AFG3L2 is highly and ubiquitously expressed in the brain. Mutations in this protein alter both m-AAA isoenzymes; in heterozygous cases, some functional overlap between these two enzymes gives mitochondria some level of functionality. Mice lacking AFG3L2 exhibit severe neurological phenotypes such as uncoordinated gait and paralysis early in their lifetimes. The partial loss of function of AFG3L2 in paraplegin-deficient mice induces more severe and accelerated phenotypes, likely due to some overlap in the activity of homo-oligomeric and hetero-oligomeric m-AAA isoenzymes. Interestingly, it appears that different neuronal types respond to these deficiencies in different ways. Purkinje cells and pyramidal hippocampal neurons, both of which express high levels of paraplegin, are hit especially hard, perhaps due to a greater dependence on the hetero-oligomeric protease (Martinelli and Rugarli, 2010; Smets et al., 2014).

Abnormalities in mitochondrial structure and function accompany m-AAA protease disruption in degenerating cells. Enlarged mitochondria with disrupted cristae accumulate in distal affected axons in paraplegin deficient mice; altered mitochondrial morphology is observed at 4.5 months, at the onset of motor impairment. HSP patients with mutant paraplegin exhibit depolarized mitochondria, reduced complex I activity, and thus reduced ATP production (Ferreirinha et al., 2004). Reduced complex I levels are also found in the brains of AFG3L2 knockout mice. Impaired axonal trafficking is also present in these cells, causing swelling and then degeneration (Maltecca et al., 2008). These changes can be reversed with rescue of paraplegin expression, as well as some degree of motor performance and neurological health (Pirozzi et al., 2014).

It appears that dysfunctional m-AAA proteases can potentially affect mitochondrial function through several different pathways, including allowing for the accumulation of misfolded proteins within mitochondria, inhibiting ATP production *via* reducing respiratory complex availability, altering mitochondrial protein synthesis *via* reducing available functional ribosomes, and affecting mitochondrial transport, though the mechanisms by which this occurs are not yet clear (Martinelli and Rugarli, 2010).

## Discussion

The entry of proteins into the mitochondria is tightly controlled, with multiple intersecting pathways ensuring that stalled peptides are removed from the mitochondrial surface, and a number of chaperones within mitochondrial spaces aiding in proper peptide folding. When import regulation fails and misfolded proteins amass within the matrix or intermembrane spaces, mitochondria activate pathways to induce greater chaperone or protease activity in an attempt to reestablish protein homeostasis. When this fails, damaged mitochondria are ubiquitinated and degraded in a process known as mitophagy.

However, we find that in several forms of neurodegeneration, these modes of quality control are compromised. Interestingly, there are commonalities between different neurodegenerative diseases, particularly among proteinopathies. Misfolded proteins interact with mitochondria, sometimes directly, and alter the energy production machinery, leading to bioenergetics defects and increased oxidative stress. Meanwhile, mitochondrial dynamics and clearance are impaired; in other words, organelle health is compromised, and dysfunctional organelles collect within the cell. It has not yet been determined whether changes to mitochondrial quality control precede protein aggregation or whether they are a cellular response to the incidence of disease; however, it is clear that the interactions between these pathways and misfolded proteins exacerbate the conditions of the cell. The buildup of protein within mitochondria inhibits degradation mechanisms, which in turn results in greater protein aggregation. This results in an increasingly worsening feed-forward loop ending in mitochondrial damage.

Several lines of inquiry are being undertaken in finding effective therapeutic strategies to combat neurodegeneration. Key features in these diseases include the induction of ROS and the failure of respiration as a result of declining mitochondrial function. Therefore, methods to reduce oxidative stress, including metabolic antioxidants and targeted antioxidants (such as Szeto-Schiller peptides), are being explored as forms of treatment for neurodegenerative symptoms, and are showing promise in early trials (Moreira et al., 2010). Treatment with antioxidants neutralizes free radicals and therefore reduces neuronal levels of oxidative stress; the high energetic needs of neurons makes them especially vulnerable to oxidative stress. Specifically, antioxidants both scavenge ROS and bind and sequester metal ions, which catalyze ROS generation (Halliwell, 1996; Chang et al., 2018). Antioxidant therapy provides an interesting avenue of exploration because it allows for both protective and restorative treatments. Dietary intake of antioxidant-rich foods can prevent protein oxidation and ROS generation, allowing for early prevention of neurodegeneration (Zandi et al., 2004). Treatment with antioxidants can rescue neurons from β-amyloid toxicity, even after plaque formation (Yao et al., 2001). Reduced inflammation as a result of antioxidant treatment has also shown promising effects in clinical trials (Arulselvan et al., 2016).

As mentioned previously, induction of mitophagy *via* small molecules such as urolithin A, rescues cellular and behavioral phenotypes in multiple neurodegenerative disease models. Interestingly, urolithin A also inhibited multiple tau phosphorylation sites in AD mouse models (Liu et al., 2018, 2022; Kujawska et al., 2020; Qiu et al., 2022). Restoration of mitophagy *via* overexpression of PINK1 was also associated with neuroprotective effects in *Drosophila* models of HD (Khalil et al., 2015). Additionally, attempts to transplant mitochondria into susceptible neurons have rescued bioenergetics defects and neuronal survival (Chang et al., 2019), suggesting a role for mitochondrial therapeutics in the future. Viability of these therapeutic strategies will rely on the effectiveness of treatment delivery as well as the ability to achieve target specificity.

## Author Contributions

AJ summarized the literature and drafted the manuscript. XQ edited the manuscript. Both authors contributed to the article and approved the submitted version.

## Conflict of Interest

The authors declare that the research was conducted in the absence of any commercial or financial relationships that could be construed as a potential conflict of interest.

## Publisher’s Note

All claims expressed in this article are solely those of the authors and do not necessarily represent those of their affiliated organizations, or those of the publisher, the editors and the reviewers. Any product that may be evaluated in this article, or claim that may be made by its manufacturer, is not guaranteed or endorsed by the publisher.
